# Clinical impact of a multiplex rapid diagnostic pneumonia panel in critically ill patients

**DOI:** 10.1017/ash.2022.358

**Published:** 2023-01-09

**Authors:** Jayda N. Esplund, Alex D. Taylor, Tyler J. Stone, Elizabeth L. Palavecino, Abdullah Kilic, Vera P. Luther, Christopher A. Ohl, James R. Beardsley

**Affiliations:** 1 Department of Pharmacy, Atrium Health Wake Forest Baptist, Winston-Salem, North Carolina; 2 Section on Infectious Diseases, Department of Internal Medicine, Wake Forest University School of Medicine, Winston-Salem, North Carolina; 3 Department of Pathology, Wake Forest University School of Medicine, Winston-Salem, North Carolina

## Abstract

**Objective:**

To evaluate the clinical impact of the BioFire FilmArray Pneumonia Panel (PNA panel) in critically ill patients.

**Design:**

Single-center, preintervention and postintervention retrospective cohort study.

**Setting:**

Tertiary-care academic medical center.

**Patients:**

Adult ICU patients.

**Methods:**

Patients with quantitative bacterial cultures obtained by bronchoalveolar lavage or tracheal aspirate either before (January–March 2021, preintervention period) or after (January–March 2022, postintervention period) implementation of the PNA panel were randomly screened until 25 patients per study month (75 in each cohort) who met the study criteria were included. Antibiotic use from the day of culture collection through day 5 was compared.

**Results:**

The primary outcome of median time to first antibiotic change based on microbiologic data was 50 hours before the intervention versus 21 hours after the intervention (*P* = .0006). Also, 56 postintervention regimens (75%) were eligible for change based on PNA panel results; actual change occurred in 30 regimens (54%). Median antibiotic days of therapy (DOTs) were 8 before the intervention versus 6 after the intervention (*P* = .07). For the patients with antibiotic changes made based on PNA panel results, the median time to first antibiotic change was 10 hours. For patients who were initially on inadequate therapy, time to adequate therapy was 67 hours before the intervention versus 37 hours after the intervention (*P* = .27).

**Conclusions:**

The PNA panel was associated with decreased time to first antibiotic change and fewer antibiotic DOTs. Its impact may have been larger if a higher percentage of potential antibiotic changes had been implemented. The PNA panel is a promising tool to enhance antibiotic stewardship.

Lower respiratory tract infections, including pneumonia, are the fourth leading cause of death worldwide, with ∼19% of pneumonia hospitalizations involving intensive care unit (ICU) admission.^1,2^ Respiratory samples are commonly obtained in the diagnostic workups of pneumonia and are utilized to guide antibiotic therapy. Identification and susceptibility testing results from respiratory cultures may take up to 72 hours. During this time, patients often remain on broad-spectrum antibiotics, increasing the risk of developing adverse events or antibiotic resistance.^3^ Pathogen identification in patients with pneumonia allows for targeted antibiotic therapy and has been associated with reduced mortality.^4^

The use of rapid diagnostic tests in healthcare settings has increased in recent years due to their timely pathogen identification, which has allowed for a decrease in broad-spectrum antibiotic use, quicker time to adequate therapy, and decreased healthcare costs.^5,6^ The BioFire FilmArray Pneumonia Panel (PNA panel, bioMerieux, Durham, NC) uses multiplex polymerase chain reaction (PCR) technology to identify 18 bacterial and 8 viral pathogens as well as 7 antibiotic resistance genes from respiratory specimens within 75 minutes of testing.^7^ Previous studies have shown the overall percentage of positive agreement between the PNA panel and respiratory cultures to be 90%–96.8% and the negative agreement to be 96.8%–98.1%.^8–10^ Our institution implemented the PNA panel in November 2021. Rather than the test being ordered by a provider, our microbiology laboratory automatically tests all respiratory specimens from adult ICU patients with quantitative cultures obtained by bronchoalveolar lavage (BAL) or tracheal aspirate (TA) with the PNA panel unless they had a PNA panel test within the preceding 72 hours. Theoretically, the more rapid identification of potential pathogens and key resistance markers would promote earlier tailoring of antibiotic therapy compared to using standard respiratory cultures alone. Although several studies have demonstrated the potential impact of the PNA panel on antibiotic therapy,^8–11^ the actual effects of the test are less well established. Therefore, we evaluated the clinical impact of the PNA panel at our institution.

## Methods

This retrospective, single-center, pre-post cohort study was approved by the institutional review board at an 885-bed academic medical center in the southeastern United States where it was conducted. This study included 2 cohorts based on date of respiratory specimen collection: a preintervention cohort (January 1–March 31, 2021) and a postintervention cohort (January 1–March 31, 2022). The following ICUs were included in the study: medical (including oncology), neuroscience, surgical, cardiothoracic, cardiovascular, trauma, and burn. During the 2-month implementation period, educational sessions and written communication were provided to critical care providers and pharmacists in these ICUs. Education included a description of pathogens and resistance genes detected by the PNA panel, the location of results in the electronic health record (EHR), and instruction on how to interpret and apply the results to guide therapeutic management. No targeted stewardship interventions were implemented with the initiation of the PNA panel. However, as with all microbiologic data, PNA panel results were reviewed daily by critical care clinical pharmacists who were available from 07:00 to 22:00 and who routinely participate in clinical decision making regarding their patients’ antimicrobial therapy.

Because we sought to determine the overall impact of the PNA panel as used at our institution, we evaluated the entire population of patients meeting criteria for the PNA panel, whether or not they had pneumonia. Patients were included in the study if they met the following criteria: age ≥ 18 years, admission to an adult ICU, and collection of a quantitative specimen obtained by BAL or TA. Patients with another bacterial infection treated inpatient or outpatient with antibiotics (other than bacteremia with the same causative pathogen) in the 14 days prior to specimen collection through 5 days after specimen collection, those who died within 5 days after specimen collection, and those who had another quantitative culture obtained by BAL or TA within the previous 72 hours were excluded.

A query of the microbiology laboratory information system identified patients admitted to an adult ICU with a quantitative BAL or TA sample obtained during the preintervention and postintervention periods. These patients were retrospectively reviewed in random order until 25 patients who met study criteria from each study month (75 patients in each cohort) were identified based on the calculation that 141 total patients would be required to detect a median difference of 30 hours between cohorts. Patients were included only once per admission.

The primary outcome was the time to antibiotic change based on microbiologic test results within 5 days of specimen collection. Secondary outcomes included potential and actual antibiotic changes made based on culture and PNA panel results, time to adequate therapy in patients who were not on adequate antibiotic therapy, number of occurrences in which therapy that was de-escalated based on the PNA panel results was re-escalated based on culture results, correlation of the PNA panel results with culture results, days of antibiotic therapy (DOT), and vancomycin serum concentration monitoring. Hospital and ICU length of stay up to 30 days from the date of culture and in-hospital mortality within 30 days of culture were also compared between the 2 cohorts. De-escalation was defined as modification of antibiotic therapy by discontinuing antibiotics or changing to an agent with a narrower spectrum of activity. Escalation was defined as switching to an antibiotic that was active against a pathogen detected that was not covered by the patient’s previous antibiotic regimen. Adequate therapy was defined as the receipt of antibiotic therapy with in vitro activity against all identified pathogens. Antibiotics were determined suitable for change if the PNA panel detected an untreated organism requiring escalation of therapy or did not detect an organism such that therapy could be de-escalated (eg, discontinuing anti-MRSA therapy if no MRSA was detected or de-escalating gram-negative therapy if no *Pseudomonas* was detected). Regarding the correlation between culture and PNA panel results, an exact correlation was defined as an exact match between pathogens identified on respiratory tract culture and those identified on PNA panel results (eg, only MRSA growing on culture, only MRSA identified on PNA panel). A sample with no growth on culture and a negative PNA panel result was also considered an exact match. Antibiotics included in DOT calculations included all systemic antibiotics administered during the 5 days following specimen collection.

The Kaplan-Meier method was used to the analyze time-to-event outcomes. If there was no antibiotic change within 5 days of obtaining the respiratory specimen, the patient was considered to have had no change, and data were censored. The χ^2^ test was used to analyze categorical data and the Mann-Whitney U test was used to analyze continuous data.

## Results

**Figure 1.**
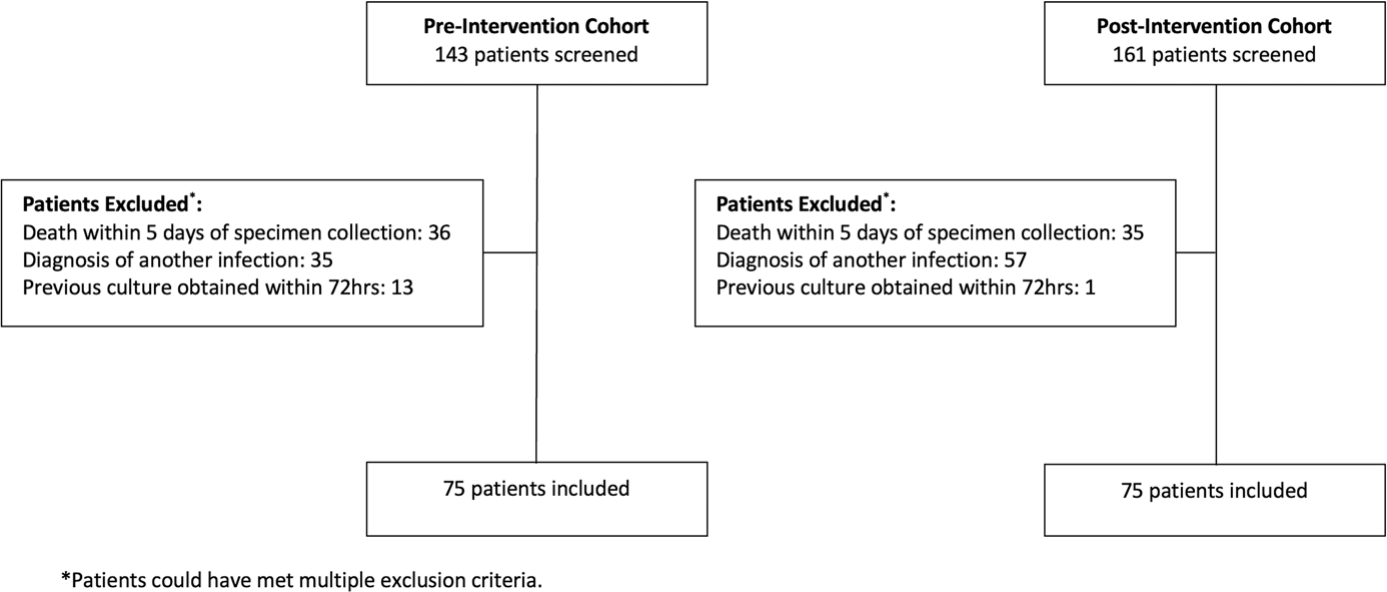
Patient Screening.

Of the 304 patients screened for inclusion in the preintervention and postintervention cohorts, 68 and 86 patients, respectively, were excluded to obtain 75 patients in each cohort who met the study criteria. The most common reasons for exclusion were death within 5 days of specimen collection and diagnosis of another infection requiring antibiotic treatment (Fig. 1). Baseline patient characteristics were similar between the 2 cohorts (Table 1). The medical and neuroscience ICUs were the most common services in both cohorts.

**Table 1.** Patient and Test Characteristics

Characteristic	Preintervention Cohort(N = 75), No. (%)^a^	Postintervention Cohort (N = 75), No. (%)^a^	*P* Value
Age, median y (IQR)	54 (44–65)	59 (38–71)	.70
Sex, female	42 (56)	32 (43)	.10
**ICU service**			.70
Medical	33 (44)	37 (49)	
Neuroscience	20 (27)	15 (20)	
Surgical	11 (15)	2 (3)	
Cardiovascular	6 (8)	2 (3)	
Cardiothoracic	2 (3)	4 (5)	
Trauma	3 (4)	12 (16)	
Burn	0	3 (4)	
Presence of chronic tracheostomy at time of culture	1 (1)	6 (8)	.05
Type of culture specimen	BAL: 11 (15) TA: 64 (85)	BAL: 10 (13) TA: 65 (87)	.81
Pathogen identified on culture	28 (37)	28 (37)	1
Polymicrobial result on culture	5/28 (18)	9/28 (32)	.22
Concurrent bacteremia	1/75 (1)	2/75 (3)	.56
COVID-19 test ordered within defined time^b^	57 (76)	62 (83)	.31
COVID-19 test positive	10/57 (18)	18/62 (29)	.14
**Pathogen identified on PNA panel**	N/A		N/A
Bacteria only		36 (48)	
Virus only		3 (4)	
Bacteria and virus		3 (4)	
**Resistance genes identified**	N/A	10 (13)	N/A
MecA/C and MREJ		8 (11)	
CTX-M		2 (3)	

Note. IQR, interquartile range; ICU intensive care unit; PNA panel: BioFire FilmArray Pneumonia Panel; BAL, bronchoalveolar lavage; TA, tracheal aspirate.

a All data represented as no. (%) unless otherwise noted.

b Defined time: 14 d before hospital admission until 5 d after culture collection.

**Figure 2.**
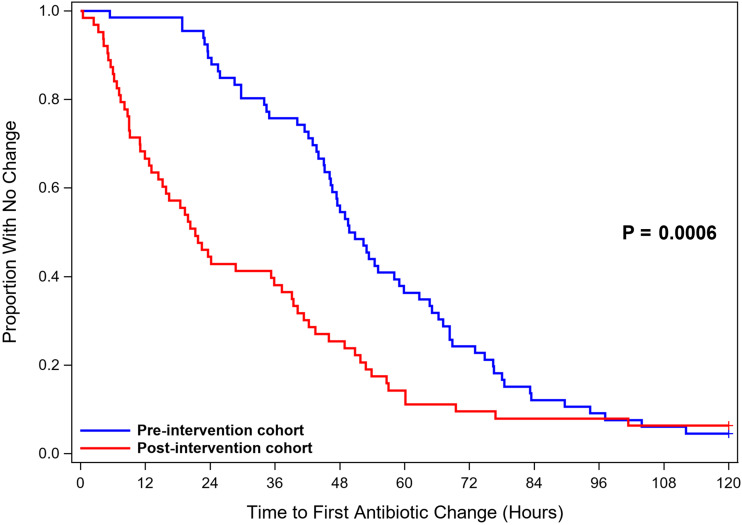
Time to Antibiotic Change.

Time-to-event outcome analyses are reported in Table 2 and shown in Figure 2. The median time to antibiotic change based on microbiologic test results was shorter in the postintervention cohort (21 hours) compared to the preintervention cohort (50 hours; hazard ratio [HR], 2.31; 95% CI, 1.57–3.39; *P* = .0006). Initial antibiotic therapy was deemed inadequate for 4 patients in the preintervention cohort and 6 patients in the postintervention cohort. The median time to antibiotic change to provide adequate therapy in this subset of patients was shorter in the postintervention cohort (37 hours) than in the preintervention cohort (67 hours; *P* = .27). In 8 instances, antibiotics were never initiated in the preintervention cohort, and in 12 instances antibiotics were never initiated in the postintervention cohort.

**Table 2.** Time to Antibiotic Change

End Point	Preintervention Cohort (N = 75)	Postintervention Cohort (N = 75)	*P* Value
Time to first antibiotic change based on microbiologic data (all patients), median h (IQR)	50 (41–69)	21 (9.0–39)	.0006
Patients treated empirically with inadequate therapy, no. (%)	4 (5)	6 (8)	.51
Time to adequate therapy in patients who were not on adequate antibiotic therapy, median h (IQR)	67 (55–70)	37 (19–48)	.27

Note. IQR, interquartile range.

In the postintervention cohort, 56 (75%) antibiotic regimens were suitable for change based on the PNA panel results. Actual changes occurred in 30 (54%) of these regimens. Of the antibiotic regimens that were changed based on the PNA panel, the median time to change was 10 hours (IQR, 7–16). Antibiotic de-escalation occurred in 23 cases, with vancomycin discontinuation being the most common change. Antibiotics were initiated in 7 cases, and gram-negative antibiotic coverage was initiated in 6 of these cases (Table 3). In 15 cases, antibiotic regimens that were not changed based on the PNA panel results were subsequently changed based on the culture results. There were no cases in which therapy that was de-escalated based on PNA panel results was later escalated based on culture results. The PNA panel was associated with decreased median total DOTs, with 8 in the preintervention cohort versus 6 in the postintervention cohort (*P* = .07).

**Table 3.** Potential and Actual Antibiotic Regimen Changes Based on PNA Panel and Culture Results in the Post-Intervention Cohort

Antibiotic Changes Based on PNA Panel	Postintervention Cohort (N = 75), No. (%)
Antibiotic regimens eligible for change	56 (75)
**Eligible antibiotic regimens changed**	30/56 (54)
De-escalation^a^	23/30 (77)
Discontinued MRSA coverage	19/23 (83)
Discontinue MRSA + gram-negative coverage	2/23 (9)
Discontinued MRSA + de-escalated gram-negative coverage	2/23 (9)
Escalation^b^	7/30 (23)
Gram-negative coverage added	6/7 (86)
Gram-positive coverage added	1/7 (14)
**Eligible antibiotic regimens not changed**	26/56 (46)
Regimens subsequently changed based on culture	15/26 (58)
Regimens not changed based on culture	11/26 (42)

Note. PNA panel, BioFire FilmArray Pneumonia Panel; MRSA, methicillin-resistant *Staphylococcus aureus*.

a De-escalation was defined as modification of antibiotic therapy by discontinuing antibiotics or changing to an agent with a narrower spectrum of activity.

b Escalation was defined as switching to an antibiotic that was active against a pathogen detected that was not covered by the patient’s previous antibiotic regimen.

The most common pathogens identified by the PNA panel were *Staphylococcus aureus* (n = 10), *Pseudomonas aeruginosa* (n = 7), and *Klebsiella pneumoniae* (n = 7). An exact correlation between the PNA panel and culture results was found in 45 samples (60%). In 14 instances, the PNA panel identified organisms that grew on culture plus additional organisms that did not grow out on culture. In 11 cases, the PNA panel identified organisms at a low concentration that did not subsequently grow on culture. In 5 instances, organisms not included in the PNA panel grew on culture: *Stenotrophomonas maltophilia* (n = 2), *Providencia stuartii* (n = 2), and *Ewingella americana* (n = 1). In no case was an organism identifiable by the PNA panel identified by culture but not by the PNA panel. No statistically significant differences in ICU length of stay, hospital length of stay, or in-hospital mortality were detected between the 2 cohorts (Table 4).

**Table 4.** Additional Secondary Outcomes

Secondary Outcomes	Preintervention Cohort (N = 75), Median (IQR)^a^	Postintervention Cohort (N = 75), Median (IQR)^a^	*P* Value
Total DOT	8 (4–9)	6 (3–9)	.07
Vancomycin DOT	2 (0–3)	1 (0–3)	.13
Antipseudomonal β-lactam, DOT	3 (0–5)	3 (0–4)	.73
Vancomycin ordered, no. (%)	48 (64)	50 (66.7)	.86
Vancomycin serum concentrations^b^	1 (0–3)	0 (0–1)	.03
ICU length of stay up to 30 days from date of culture^b^	5.6 (3–13)	7.1 (4–10)	.37
Hospital length of stay up to 30 d from date of culture, d^b^	11.3 (6–17)	12.9 (8–23)	.09
In-hospital mortality within 30 d from date of culture, no. (%)	17 (23)	17 (23)	1

Note. IQR, interquartile range; DOT, days of antibiotic therapy; ICU, intensive care unit.

a All data represented as median (IQR) unless otherwise noted.

b Per person in each cohort.

## Discussion

Current pneumonia guidelines recommend using respiratory cultures to guide targeted antibiotic therapy.^12,13^ Most patients with suspected hospital-acquired or ventilator-associated pneumonia are initiated on a combination of empiric broad-spectrum antibiotics, often vancomycin and an antipseudomonal β-lactam.^14^ Once results are available, antibiotic therapy should be narrowed to the most effective agent against the causative pathogen(s). Rapid diagnostic tests can provide accurate and early pathogen identification, decreasing the time to targeted therapy compared to using traditional culture data alone.^9,10^ Although many studies have been conducted to determine the potential impact of the PNA panel, data on its actual impact in clinical practice are limited. In this study, we sought to obtain real-world data on the impact of the PNA panel at our institution.

The PNA panel significantly decreased the time to antibiotic change in critically ill patients, with the greatest impact on the de-escalation of MRSA antibiotic coverage. Furthermore, when exclusively looking at patients in whom the PNA panel result was used to prompt antibiotic change, the median time to antibiotic change was reduced to 10 hours. The use of the PNA panel was also associated with fewer days of antibiotic therapy. In our study, the PNA panel led to a quicker time to adequate therapy in patients who were initially on inadequate therapy. By facilitating a more rapid change to targeted therapy, rapid diagnostic tests, such as the PNA panel, can aid in decreasing broad-spectrum antibiotic use and decreasing the time to adequate antibiotic therapy.

Although the PNA panel had a significant impact on antibiotic optimization in adult ICUs, opportunities for improvement were identified. Although the PNA panel had the potential to influence 75% of the therapy courses, it only led to antibiotic changes in approximately half of these patients. To understand these cases further, an analysis was conducted that evaluated patients in the postintervention cohort who could have had therapy modified based on PNA panel results but did not. In approximately half of these cases, antibiotics were subsequently changed based on the culture results, which may indicate a lack of trust in the PNA panel. With more time and experience, confidence in using this new test to modify antibiotic therapy may increase. In the other half of the patients, therapy was not modified even after culture results were reported, potentially indicating a general reluctance to de-escalate therapy in these patients. The panel may also have had a greater effect if stewardship interventions beyond the actions of critical care clinical pharmacists, which occur predominantly during the day shift, had been implemented. Multiple recent studies have shown the positive effects of real-time, active methods to assist with optimizing antibiotic stewardship practices using rapid diagnostic tests.^15–17^ These efforts by antibiotic stewardship clinicians can help decrease the initial hesitancy of some providers to embrace these new technologies and assist with their appropriate application to antibiotic use.

In our study, exact agreement occurred between the PNA panel and culture results for 60% of samples, which is similar to previous studies that reported 53.6%,^8^ 46.3%,^9^ and 47.4%^18^ concordance rates. The number of discordant PNA panel and culture results were anticipated given that the number of pathogens detected by the PNA panel is not exhaustive and that the panel can detect both nonviable and viable organisms at very low quantities. Therefore, it was expected that the PNA panel would detect organisms from a respiratory sample that did not grow on culture. No instances occurred in which an organism detectable by the PNA panel was not identified by the PNA panel but subsequently grew on culture, supporting the high negative predictive value of the test. Based on these results, clinicians should feel confident in de-escalating anti-MRSA or antipseudomonal antibiotic therapy if the respective pathogens and resistance genes are not identified on the PNA panel in critically ill patients with pneumonia. As with all tests, the PNA panel is a tool that must be used in conjunction with an assessment of patient-specific factors to optimize care.

This study had several limitations. Owing to its retrospective design, the data collected were limited to those recorded in the EHR. This study was conducted at an institution that did not consistently use MRSA nasal screening to de-escalate anti-MRSA coverage. Therefore, the impact on anti-MRSA therapy utilization may not translate to an institution with more universal MRSA screening practices. As more rapid diagnostic tests become available and are used together, it may be beneficial to evaluate the impact of each test individually and when used in concert. In addition, our study was conducted during the coronavirus disease 2019 (COVID-19) pandemic, and it is unknown how the differences in severe acute respiratory coronavirus 2 (SARS-CoV-2) variants, treatments, and vaccine reception may have affected the different study groups. Also, we did not control for confounding factors, such as patient comorbidities (eg, neutropenia, immunosuppression, past infections with multidrug-resistant pathogens), severity of illness, and hemodynamic instability, which could have influenced de-escalation decisions. Finally, our postintervention cohort was studied shortly after the implementation date of the PNA panel; therefore, our results may not represent the application of this new test after providers became more familiar with its use.

In conclusion, the PNA panel was associated with a significant decrease in the time to the first antibiotic change and fewer antibiotic days of therapy. The PNA panel may have had a greater impact on antimicrobial use if more antibiotic changes had been implemented at the time of PNA panel result. The PNA panel is a promising antimicrobial stewardship tool.
